# Regulation of Monocyte-Macrophage Responses in Cirrhosis—Role of Innate Immune Programming and Checkpoint Receptors

**DOI:** 10.3389/fimmu.2019.00167

**Published:** 2019-02-05

**Authors:** Antonio Riva, Gautam Mehta

**Affiliations:** ^1^Institute of Hepatology London, Foundation for Liver Research, London, United Kingdom; ^2^Faculty of Life Sciences & Medicine, King's College London, London, United Kingdom; ^3^UCL Institute for Liver and Digestive Health, University College London, London, United Kingdom

**Keywords:** cirrhosis, ACLF, ARLD, ALD, alcoholic hepatitis, innate immune cell, myeloid, immune checkpoint

## Abstract

Many aspects of the innate immune system have been studied in cirrhosis, and abnormalities have been described supporting both a pro-inflammatory and anti-inflammatory phenotype of myeloid cells. However, the findings of these studies vary by stage of disease and methodology. The recent description of the syndrome of acute-on-chronic liver failure (ACLF) has refined our understanding of the natural history of cirrhosis. In this context, we review the regulatory mechanisms at play that contribute to the immune abnormalities described in advanced liver disease. Specifically, we review the evidence for epigenetic mechanisms regulating monocyte phenotype, and the role of checkpoint receptors on regulating innate and adaptive immune cell function.

## Background

This is an exciting time for the field of immunotherapeutics. Advances in basic science and drug development have progressed our understanding of regulatory mechanisms of both innate and adaptive immune responses, which has directly led to novel immunotherapeutic agents. Moreover, technological advances have allowed unbiased data collection from monocyte-macrophage lineage cells, allowing a deeper understanding of their diversity and plasticity (1). The purpose of this review is to integrate these data and place them within the context of the disease landscape of cirrhosis.

Inflammation, and consequently innate immunity, plays a key role in the development of liver disease at almost every stage. For example, in the early stages, monocyte-macrophage lineage cells play a role in both the development (2, 3) and the resolution (4) of hepatic fibrosis. Understanding and harnessing the mechanism of fibrosis resolution by hepatic macrophages is an area of active translational research, although is beyond the scope of this review. This area has been reviewed recently by Ramachandran et al. (5).

**Figure 1:**
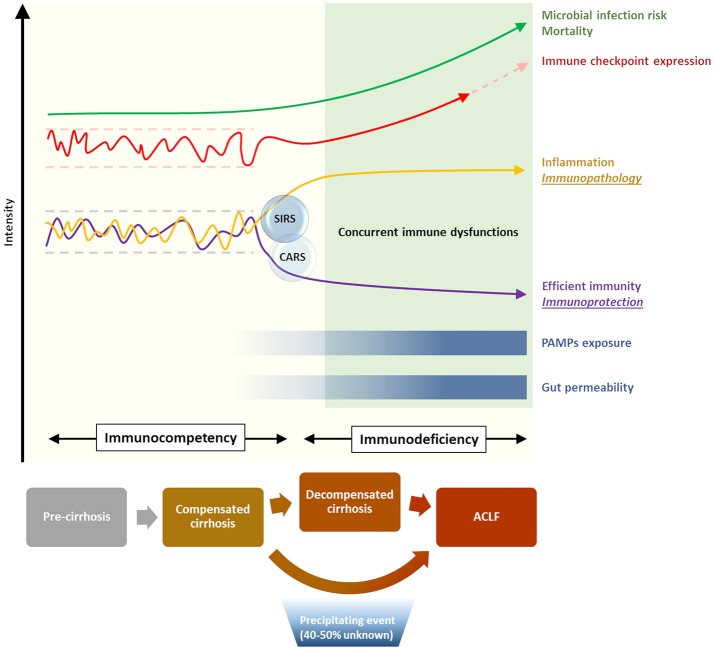
Immunological phenomena associated with progression of cirrhosis. Increasing disease severity is accompanied by the establishment of a skewed immune profile, characterized by concurrent systemic inflammation and deficient immune protection. This state of cirrhosis-associated immune dysfunction (CAID) is crucial in increasing the risk of life-threatening microbial infection, and is in part mediated by a dysregulation of the immune checkpoint network [reviewed in (9)].

Acute-on-chronic liver failure (ACLF) is a recently defined syndrome, describing an acute clinical deterioration on the background of cirrhosis, characterized by a rapid progression to multi-organ failure and high mortality. The CANONIC study, the largest prospective study of the natural history of cirrhosis, demonstrated that in the majority of cases (60%) a specific trigger for the acute deterioration, such as bacterial infection or acute alcoholic hepatitis, could be identified (6). It was also apparent from this study that dysregulated inflammation is a key feature of the syndrome. The degree of systemic inflammation, determined by leukocyte count and C-reactive protein, was an independent predictor of the development and prognosis of ACLF. However, alongside these pro-inflammatory responses, immunodeficiency and susceptibility to infection are also features of cirrhosis and ACLF (7, 8). The overarching term for these immune alterations in cirrhosis is cirrhosis-associated immune dysfunction (CAID), although the mechanisms that regulate these diverse and dichotomous immune responses in cirrhosis remain incompletely understood (Figure 1). Nevertheless, recent insights into immune pathobiology in cirrhosis, along with general advances in our understanding of regulation of immunity, provide opportunities for novel therapies in cirrhosis. These opportunities will be discussed in more detail in this review.

The lines of evidence describing both features of exaggerated systemic inflammation, as well as immunodeficiency in cirrhosis and ACLF have been recently reviewed elsewhere (7). From a mechanistic perspective, gut bacterial dysbiosis and the translocation of bacterial products to mesenteric lymph nodes and the systemic circulation have been suggested to play a role in the development of these immunological abnormalities in cirrhosis (7) (Figure 1). However, a clear association of gut dysbiosis and a specific immune phenotype has not yet been demonstrated. Moreover, most evidence from cirrhotic patients is from single time points, and from varying severities of liver disease, as prospectively collected data delineating the time-course of immune phenotype in cirrhosis and ACLF is currently lacking. As can be seen in Table 1, both pro- and anti-inflammatory responses from monocyte-macrophage cells have been described in cirrhosis. Further discussion of the evidence behind these observations is provided below.

**Table 1:** Monocyte-macrophages display pro- and anti-inflammatory phenotypes in end-stage liver disease.

**Anti-inflammatory phenotype**		**Pro-inflammatory phenotype**	
**Monocyte-Macrophage:**		**Monocyte-Macrophage:**	
Rimola et al. (10)	*In vivo:* Decreased reticuloendothelial system phagocytosis in cirrhosis (DC) compared to HC, by tracer elimination method. This was associated with increased incidence of bacterial infection.	Albillos et al. (11)	*Ex vivo:* Increased activation markers (HLA-DR and CD80) and intracytoplasmic TNFα expression in monocytes from cirrhosis (DC) compared to HC.
Gomez et al. (12)	*In vivo:* Decreased macrophage-mediated clearance of IgG-coated erythrocytes in cirrhosis (mixed SC and DC). This was associated with increased incidence of bacterial infection.	Tazi et al. (13)	*Ex vivo:* Greater increase in LPS-induced monocyte TLR4 expression and TNFα release from cirrhotic patients compared to HC.
Wasmuth et al. (14)	*Ex vivo:* Decreased monocyte LPS-induced TNFα production and HLA-DR expression in ACLF compared to SC.	Gandoura et al. (15)	*Ex vivo:* Microarray gene expression profiling of PBMCs from ARLD cirrhosis (DC) showed decreased induction of type-1 and type-2 IFN-stimulated genes, compared to HC (see left column).
Gandoura et al. (15)	*Ex vivo:* Microarray gene expression profiling of PBMCs from ARLD cirrhosis (DC) compared to HC, showed increased induction of pro-inflammatory cytokine genes (IL-6, IL-8, TNFα), but decreased induction of type-1 and type-2 IFN-stimulated genes, compared to HC (see right column).		
O'Brien et al. (16)	*Ex vivo:* Plasma from DC and ACLF led to decreased LPS-stimulated TNFα release and bacterial killing when incubated with healthy monocyte-macrophages, compared to plasma from stable cirrhosis.		
Bernsmeier et al. (17)	*Ex vivo:* Decreased monocyte LPS-induced TNFα and IL-6 production in DC and ACLF compared to stable cirrhosis. No change in ROS production.		

## Circulating Monocytes

**Figure 2:**
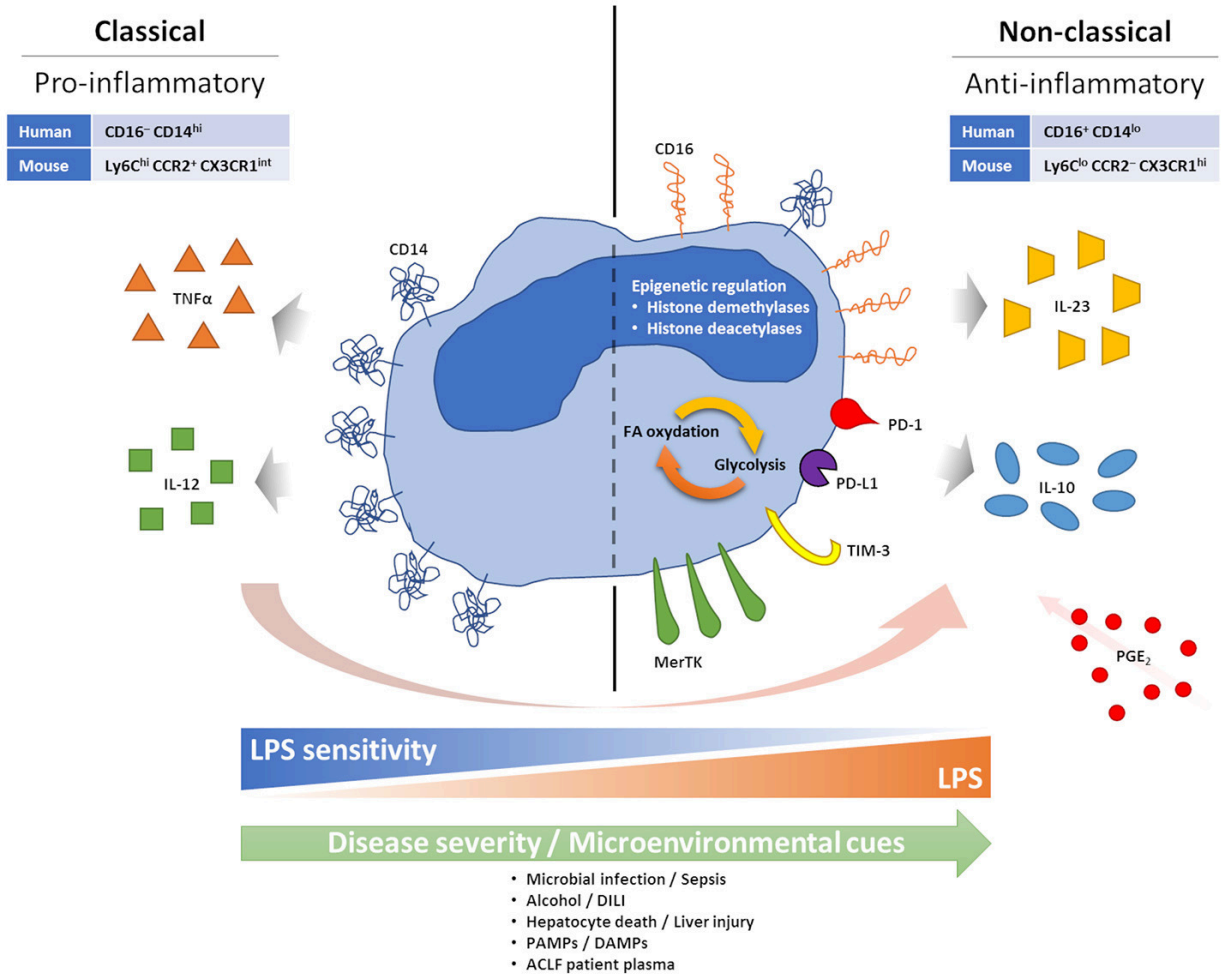
Features of anti-inflammatory monocytes. During acute excessive inflammation monocytes can acquire an anti-inflammatory phenotype. Mechanisms behind this phenomenon are not fully understood, but chronic and intense stimulation with PAMPs and DAMPs, consequent to microbial infection or tissue damage/injury, seems able to facilitate it. PGE2 also seems to play a role in enabling monocyte changes. Anti-inflammatory monocytes appear tolerised to bacterial endotoxin, possibly linked to loss of CD14. High CD16 and MerTK have also been described. Monocyte expression of inhibitory immune checkpoints has been linked to a more suppressive phenotype, with preferential IL-10 production, and worse prognosis in cancer, infections and sepsis. Epigenetic reprogramming, and its effect on intracellular metabolic pathways, are also observed in anti-inflammatory monocytes.

Circulating monocytes play an important role in host defense, through initiation and regulation of inflammatory responses (18). In both humans and mice their phenotype can be divided into two main subsets: classical (pro-inflammatory) and non-classical (anti-inflammatory, pro-repair), which are distinguished by surface markers (19, 20). These subsets are primarily separated by their expression of CD14 (the co-receptor for bacterial lipopolysaccharide, LPS) and CD16 (a low affinity type III Fc receptor for IgG). Most circulating monocytes, around 90%, are classical CD16^−^ monocytes expressing high levels of CD14 (CD16^−^CD14^+^). The remainder CD16^+^ monocytes are further separated based on the expression of CD14 among CD16^+^CD14^+^ intermediate monocytes and CD16^+^CD14^lo^ non-classical monocytes. Similar subsets are found in mice using the Ly6C, CCR2, and CX3CR1 markers, with classical Ly6C^hi^CCR2^+^CX3CR1^int^ monocytes and non-classical Ly6C^lo^CCR2^−^CX3CR1^hi^ monocytes (Figure 2).

Conceptually, our understanding of the natural history of cirrhosis has progressed over recent years with description of the syndrome of ACLF, which describes patients with cirrhosis who progress from stable or decompensated cirrhosis to a rapid decline in liver function and extra-hepatic organ failure following a superimposed “hit”. As can be seen from Table 1, there are few studies that examine immune cell phenotype in this stage of the disease. Monocyte dysfunction has been previously described in ACLF in cross-sectional studies, indicating skewed proportions between monocyte subsets with an increasing prevalence of anti-inflammatory monocytes able to suppress pro-inflammatory innate immune responses correlated with disease severity. Specifically, increased numbers of monocytes expressing the receptor tyrosine kinase Mer (MerTK) have been found in ACLF, associated with reduced pro-inflammatory responses *ex vivo* (17), and similarly, prostaglandin E2 (PGE2) levels have been found to be elevated in ACLF and implicated in the anti-inflammatory monocyte phenotype (16) (Figure 2). However, an overarching mechanism for the change in monocyte phenotype in ACLF is currently lacking.

## Circulating Monocytes Respond to Superimposed Liver Injury by Altering their Phenotype and Function

The superimposed “hit” in ACLF, on the background of cirrhosis, has been suggested to represent an acute liver insult such as gut bacterial translocation, sepsis, alcoholic hepatitis or drug-induced liver injury (DILI), leading to hepatocyte cell death and the release of damage/danger-associated molecular patterns (DAMPs) (8). Therefore, a possible hypothesis for the change in circulating monocyte phenotype in ACLF is that it represents a regulatory response to this superimposed liver injury (Figure 2).

The traditional dogma from mouse experiments has been that monocytes sequentially alter their phenotype from classical to non-classical over time, possibly in response to micro-environmental cues. For example, following injury, predominantly Ly6C^hi^ monocytes are recruited from the bone marrow and spleen to sites of injury in a CCR2- and CCL2-dependent manner (21–23). Recent elegant experiments using deuterium labeling in humans, and adoptive transfer experiments in humanized mice, has demonstrated similar transitioning in human monocytes, particularly in response to challenge with bacterial endotoxin (24). The time course for this transition from classical to non-classical phenotype was between 1 and 5 days, with non-classical monocytes persisting for around 12 days, thus demonstrating the importance of time course in determining immune phenotype following infection or injury (Figure 2).

The relevance of these observations to liver injury has also been explored in rodent models. In a model of acetaminophen (APAP)-induced liver injury, fate-mapping studies using adoptive transfer of Ly6C^hi^CX3CR1^+^ monocytes demonstrated that these Ly6C^hi^ monocytes differentiated into a Ly6C^lo^ subset by 72 h following liver injury, and were cleared by 96 h (25). A further study by Dal-Secco and colleagues used CCR2-RFP and CX3CR1-GFP double-reporter mice with a model of sterile liver injury (26). These elegant experiments demonstrated that CCR2^hi^CX3CR1^lo^ (Ly6C^hi^) monocytes were initially recruited to the site of liver injury, and over a period of 24 h transitioned into a CCR2^lo^CX3CR1^hi^ (Ly6C^lo^) subset that was prevalent for up to 72 h. Therefore, it is clear that liver injury can influence the phenotype of infiltrating cells, but it remains unclear whether this occurs in cirrhosis, and to what extent this influences the phenotype of circulating immune cells. Nevertheless, most studies of monocytes in ACLF have demonstrated that monocyte dysfunction can be induced in healthy monocytes by incubation with ACLF plasma (16, 17) (Figure 2). Therefore, liver injury may lead to the subsequent reprogramming of circulating cells as well as infiltrating monocytes to an anti-inflammatory phenotype—this hypothesis merits further attention.

## De novo Recruitment of Anti-Inflammatory Cells

A further possible mechanism is the *de novo* recruitment of anti-inflammatory cells. In humans, a recent study in ACLF described an increased population of circulating CD14^+^CD15^−^HLA-DR^−^ myeloid-derived suppressor cells, which lead to impaired innate and adaptive immune responses and thus contribute to the anti-inflammatory phenotype of ACLF (27).

Another tier of complexity is the possible infiltration of peritoneal-derived macrophages following liver injury. Recent elegant work from Paul Kubes' lab has demonstrated that these peritoneal cells, described as F4/80^+^CD11b^hi^CD102^+^ and GATA6^+^, were found to relocate to the liver within 1 h following liver injury, to express markers associated with tissue repair, and to be critical for survival of mice following CCl4-induced acute liver injury (28). Furthermore, using CCR2-RFP and CX3CR1-GFP double-reporter mice, it was clear that these peritoneal macrophages are distinct from infiltrating peripheral blood monocytes. Further work is required to see if these cells are present in humans, and to what extent (if any) they can modify systemic immune phenotype.

## Monocyte Reprogramming and Epigenetics

In a broader sense, the molecular mechanisms of monocyte reprogramming are beginning to be understood and exploited. The concept of “innate immune memory” has arisen over recent years, challenging the dogma that only adaptive immune cells have the capacity for “memory” (29). This concept describes the phenomenon whereby an innate immune cell can mount a qualitatively different response, either exaggerated (“trained immunity”) or impaired (tolerance), in response to repeated challenge. As such, it is becoming clear that innate immune cells, particularly monocytes, can be reprogrammed at metabolic, epigenetic, and transcriptional levels (30). In situations with acute excessive inflammation, tolerance acts as a mechanism to dampen the inflammatory response of the host and maintain homeostasis to prevent tissue damage and organ failure (31, 32). Nevertheless, in conditions such as sepsis, chronic inhibitory effects in immune function can also lead to a state of deep and long-lasting immunosuppression associated with a higher risk of secondary infections and a poorer outcome (33).

Epigenetic mechanisms have been implicated in monocyte-macrophage reprograming (30) (Figure 2). In response to LPS or upon pathogen exposure, monocytes and macrophages modify their histone acetylation and methylation marks, affecting gene expression patterns upon subsequent stimulation (34). For example, after LPS exposure, the repressive histone modification “H3K9 dimethylation” (H3K9me2) is noted at the promoter regions of IL-1β and TNFα (35, 36). Potential molecular mechanisms include increased expression of histone demethylases and deacetylases following LPS exposure (37, 38), and several of these mechanisms have also been involved in the reprogramming of intracellular metabolic activities affecting the balance between glycolysis and fatty acid oxidation (39, 40) (Figure 2). These pathways are potentially targetable: inhibitors of the histone deacetylases sirtuin 1 and 2 (SIRT1/2) have shown efficacy in reversing immune paralysis in mouse models of sepsis (41, 42). Similarly, long non-coding RNAs have been shown to be mediators of a “switch” in monocyte phenotype in sepsis and are also potentially targetable through antisense nucleotide strategies (43). These mechanisms deserve attention in cirrhosis and ACLF.

## Immune Checkpoints

A further level of regulation is through interaction with adaptive immune cells and regulation of signaling through immune checkpoint receptors. Immune checkpoints constitute a complex array of regulatory receptors and ligands that are expressed on the surface of both innate and adaptive immune cells. Both co-stimulatory and a relatively larger set of inhibitory checkpoint pathways have been described, and it is the fine balance between all these positive and negative signals that is responsible for the physiological regulation of the fate and direction of ongoing immune responses. The expression of these regulatory pathways is both anatomically and temporally coordinated in order to facilitate the initiation and the termination of immune responses. However, in situations where the inflammation or the antigenic stimulation persist (such as sepsis, endotoxemia, or chronic infections) inhibitory checkpoints remain upregulated and this overwhelming negative signaling leads to immune cell exhaustion and immunosuppression. Amongst the most characterized inhibitory checkpoints, PD-1 and CTLA4 (with their respective ligands PD-L1 and CD80) have demonstrated to be novel, effective and safe immunotherapeutic targets for cancer, and new monoclonal blocking antibodies for TIM-3 are also currently in clinical development or tested in clinical trials [reviewed in (9)].

Most checkpoint pathways have been first characterized as regulators of T-cell immunity, but it is now clear that their effects are not limited to T cells only. For instance, PD-1 is known to also cause B and NK cell functional suppression [reviewed in (9)], and a study in HIV patients demonstrated that monocytes can express PD-1 upon bacterial exposure. These PD-1^+^ monocytes secrete suppressive IL-10 upon PD-1 engagement (Figure 2), and either PD-1 or IL-10 receptor blockade in these patients can reverse adaptive HIV-specific T-cell exhaustion(44) (Figure 2). Expression of PD-1 and PD-L1 on monocytes has also been associated with increased mortality in septic patients (45, 46), while expression of TIM-3 on monocytes has been linked to a more aggressive tumor phenotype in gastric cancer patients (47), a reduced pathogen clearance in malaria (48), preferential production of IL-10 and suppression of IFNγ T-cell responses in osteosarcoma patients (49). Furthermore, it has been proposed that expression of TIM-3 on monocytes may be able to shift the balance from IL-12 to IL-23 production and consequently favor type-17 rather than type-1 T-cell responses, driving IL-17-mediated inflammation at the expense of anti-pathogen IFNγ-mediated responses (50, 51) (Figure 2). Monocyte expression of TIM-3 is further inducible in response to TLR agonists, including TLR4-mediated LPS stimulation, and this can have a relevant impact in defining the immune milieu in response to bacteria or viruses (50, 51). Importantly, blockade of monocyte TIM-3 seems able to reverse the majority of these regulatory or suppressive effects, supporting the restoration of effective immune responses (48, 49, 51–53)

The above-described altered landscape of immunity in advanced liver disease is influenced by checkpoint receptor expression. A paper published in 2015 by one of the authors (AR) was the first to demonstrate that PD-1 and TIM-3 are key in defining this altered immune landscape, and monocyte hyper-stimulation with gut-derived bacterial LPS was found to be the driving factor for these immune dysfunctions (54). We observed that adaptive antibacterial T-cell responses in patients with advanced alcohol-related liver disease were prominently skewed toward the production of suppressive IL-10 in response to LPS stimulation, and this was directly correlated with loss of IFNγ production and hyper-expression of PD-1 and TIM-3 on several immune cell subsets, including T, NK, and NKT cells (54), but not—interestingly—innate-like antibacterial T cells (mucosal-associated invariant T cells, or MAIT) (55). Stimulation with LPS dose-dependently induced PD-1, TIM-3 and IL-10 expression, but blockade of TLR4 and CD14 on monocytes completely abolished these effects; furthermore, blocking PD-1 and TIM-3 suppressed IL-10 and restored the production of antibacterial IFNγ, indicating that the immune defects observed in patients with alcohol-related liver disease may be reversible (54). Similar findings have been described in patients with non-alcoholic sepsis and also in mouse models of sepsis-induced endotoxin-driven liver inflammation [reviewed in (9)].

These results indicate that immune checkpoint blockade may be an effective treatment strategy for the restoration of defective antibacterial immunity in patients with end-stage liver disease. Furthermore, the lack of inflammation observed in our study and the good safety profiles of anti-checkpoint monoclonal antibodies currently used in cancer and sepsis suggest that immune checkpoint blockade may be a safe treatment approach also in end-stage liver disease, where conventional treatment options are currently very limited.

## Conclusion

In conclusion, the innate and adaptive immune systems have many tiers of regulation which have been shown to be dysfunctional in cirrhosis. However, prospectively collected data delineating the time course of immune phenotype by stage of disease in cirrhosis remains scarce. Innate cell reprogramming, through metabolic or epigenetic mechanisms or by targeting checkpoint receptors, remains an attractive area for translational development, although parallel development of reliable immune biomarkers in cirrhosis will be required for immunotherapies to reach their full potential.

## Author Contributions

All authors listed have made a substantial, direct and intellectual contribution to the work, and approved it for publication.

### Conflict of Interest Statement

The authors declare that the research was conducted in the absence of any commercial or financial relationships that could be construed as a potential conflict of interest.

## Abbreviations

ACLF: Acute-on-chronic liver failure
AH: Alcoholic hepatitis
ARLD: Alcohol-related liver disease
CD: Cluster of differentiation
CTLA-4: Cytotoxic T-lymphocyte associated protein 4
DAMP: Damage/Danger-associated molecular pattern
DC: Decompensated cirrhosis
IFN: Interferon
IL: Interleukin
MAIT: Mucosal-associated invariant T cells
NK: Natural killer cells
NKT: Natural killer T cells
PAMP: Pathogen-associated molecular pattern
PD-1: Programmed death 1
PD-L1: Programmed death ligand 1
SC: Stable cirrhosis
TIM-3: T-cell immunoglobulin and mucin domain 3 (also known as Hepatitis A virus cellular receptor 2, HAVCR2)
TNF: Tumor necrosis factor
